# Genetic Variation in the Catechol-O-Methyl Transferase Val108/158Met Is Linked to the Caudate and Posterior Cingulate Cortex Volume in Healthy Subjects: Voxel-Based Morphometry Analysis of Brain Magnetic Resonance Imaging

**DOI:** 10.1371/journal.pone.0142862

**Published:** 2015-11-13

**Authors:** Keita Watanabe, Shingo Kakeda, Reiji Yoshimura, Satoru Ide, Kenji Hayashi, Asuka Katsuki, Wakako Umene-Nakano, Rieko Watanabe, Osamu Abe, Yukunori Korogi

**Affiliations:** 1 Department of Radiology, University of Occupational and Environmental Health, Fukuoka, Japan; 2 Department of Psychiatry, University of Occupational and Environmental Health, Fukuoka, Japan; 3 Department of Radiology, Nihon University School of Medicine, Tokyo, Japan; UTHSCSH, UNITED STATES

## Abstract

The effect of the catechol-O-methyltransferase (COMT) Val158Met polymorphism on brain morphology has been investigated but remains controversial. We hypothesized that a comparison between Val/Val and Val/Met individuals, which may represent the most different combinations concerning the effects of the COMT genotype, may reveal new findings. We investigated the brain morphology using 3-Tesla magnetic resonance imaging in 27 Val/Val and 22 Val/Met individuals. Voxel-based morphometry revealed that the volumes of the bilateral caudate and posterior cingulate cortex were significantly smaller in Val/Val individuals than in Val/Met individuals [right caudate: false discovery rate (FDR)-corrected p = 0.048; left caudate: FDR-corrected p = 0.048; and bilateral posterior cingulate cortex: FDR-corrected p = 0.048]. This study demonstrates that interacting functional variants of COMT affect gray matter regional volumes in healthy subjects.

## Introduction

Catechol-O-methyltransferase (COMT) is a methylation enzyme that facilitates dopamine degradation by catalyzing the transfer of a methyl group from S-adenosylmethionine to dopamine in the extrasynaptic spaces. The COMT gene resides in the q11 region of chromosome 22 [1], where a functional missense mutation causes a single G-to-A base-pair substitution, resulting in a single nucleotide polymorphism in exon 4. This polymorphism changes codons 108/158 from methionine (Met) to valine (Val) [2]. Met/Met individuals have a 4-fold decrease in enzymatic activity relative to Val/Val individuals, resulting in higher dopamine concentrations in the extrasynaptic space [2]. Val/Met individuals exhibit intermediate levels of enzymatic activity compared with that of Val/Val and Met/Met individuals [3].

Because dopamine plays a critical role in synaptic plasticity, it would be expected that the COMT genotype may also exert an important influence on the brain morphology of healthy subjects. Some investigators have found that Met alleles are associated with increased measures of volume/cortical thickness in the frontal and temporal cortex [4, 5] or posterior cingulate cortex [6]. On the other hand, Cerasa et al. and Honea et al. reported that Val alleles were associated with increased volume in the frontal cortex, whereas Met alleles are associated with increased volume in the hippocampus [7, 8]. These studies using the whole brain analysis support the hypothesis that the COMT genotype affects the brain morphology, although the relationship between affected brain area and the COMT genotype were conflicting. In the contrary, Barnes et al. and Ohnishi et al. did not find the statistically significant association between gray matter (GM) volume and the COMT genotype in the whole brain analysis [9, 10]. The studies using region of interest (ROI) analysis also demonstrated that the COMT genotype showed no significant relationship with the frontal cortex [11] and hippocampal volume [12]. Thus, the relationship between the COMT genotype and brain morphology is controversial. Differences in not only the image analysis methods but also the study designs may explain the discrepancies in these previous reports [4–9, 12]. Many previous investigators assessed the COMT genotype effect using the comparison between Val/Val individuals versus Met carriers (Val/Met and Met/Met) or Val carriers (Val/Val and Val/Met) versus Met/Met individuals [6–8, 10] or the linear regression model (i.e., Val/Val = 1, Val/Met = 2, and Met/Met = 3) [4, 5, 7, 9]. However, there was a few studies which assessed the difference between each COMT genotype (Val/Val, Val/Met, and Met/Met) using the t-tests [7] or factorial analysis of variance in which the COMT genotype was set as a factor [6].

**Fig 1:**
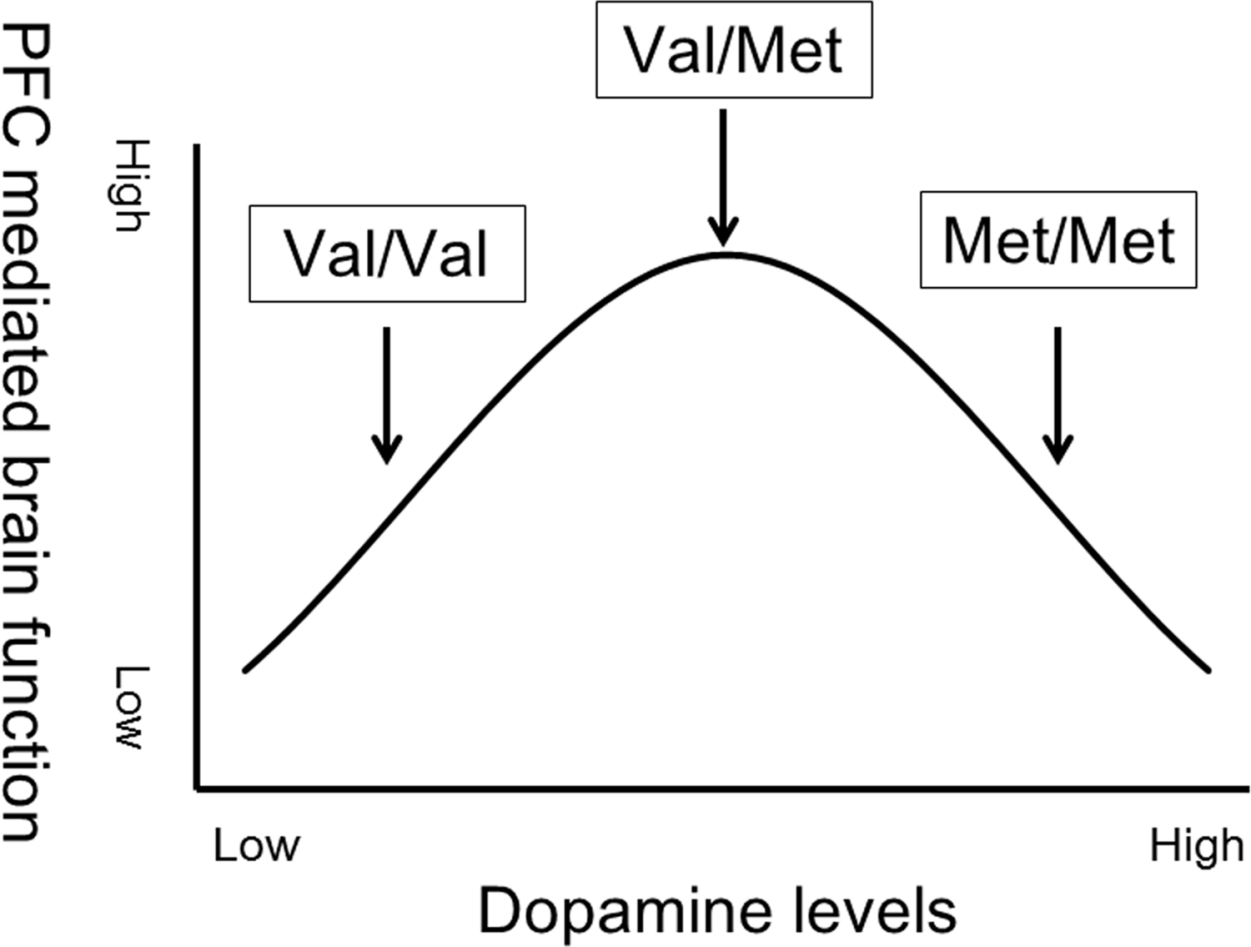
Hypothetical inverted U-shaped relationship between prefrontal cortex (PFC) brain function and dopamine levels. Intermediate COMT activity and dopamine levels present in Val/Met individuals are thought to be optimal for PFC-mediated brain function.

The relationship between dopamine levels and brain function/morphology is complicated. There appears to be an inverted U-shaped dose-response curve by which both deficient and excessive amounts of dopamine activity predict poor performance on cognitive tasks [13–15]. Preclinical data has suggested that these functional effects are related to a lawful effect of extracellular dopamine stimulation on the firing rate and tuning of prefrontal neurons, which exhibit optimal function with a middle range of dopamine [13, 16]. A previous study that assessed the relationship between COMT genotypes and cognitive tasks in adolescents also suggested that Val/Met individuals, who have intermediate COMT activity and dopamine levels, displayed optimal performance [17] (Fig 1).

Based on the results of these studies, we hypothesized that the comparison of Val/Val and Val/Met individuals, which are considered to be the most different among the COMT genotypes, may reveal a new finding. The purpose of this study was to identify specific brain regions with morphological differences correlated with the COMT Val108/158Met genotype using a voxel-based morphometry (VBM) analysis of brain magnetic resonance imaging (MRI) data.

## Methods

### Participants

The protocol of this prospective study was approved by the Ethics Committee of the University of Occupational and Environmental Health. All participants provided written informed consent prior to study participation.

Fifty-two healthy subjects were recruited via an interview conducted by a psychiatrist (K.H. with 7 years of experience in psychiatry) using the Structured Clinical Interview for DSM-IV, non-patient edition. No subjects had a history of serious medical or neuropsychiatric illness or a family history of major psychiatric or neurological illness among their first-degree relatives.

### Genotyping

Fifty-two subjects provided a blood sample from which DNA was extracted according to standard laboratory protocols. These samples were genotyped for the COMT Val108/158Met polymorphism using direct regional sequencing. In brief, DNA was isolated from peripheral blood mononuclear cells using the QIAamp DNA Mini-Kit (QIAGEN, Tokyo, Japan). Genotyping was performed with a polymerase chain reaction single nucleotide polymorphism (SNP) genotyping system using the BigDye Terminator v3.1 Cycle Sequencing Kit (Life Technologies Japan, Tokyo, Japan). The DNA sequence was read using a BMG Applied Biosystem 3730xI DNA Analyzer (Life Technologies Japan, Tokyo, Japan). We used a forward primer (TCACCTCTCCTCCGTCCCAA) and a reverse primer (ACAAGGCCCCCACTCTGTCCCT) specific for the COMT Val108/158Met polymorphism.

The 52 healthy subjects were divided based on their COMT genotype: 27 with the Val/Val genotype, 22 with the Val/Met genotype, and 3 with the Met/Met genotype. We excluded the 3 Met/Met individuals and compared brain morphology between Val/Val and Val/Met individuals.

### Magnetic Resonance Imaging and Image Processing for Voxel-Based Morphometry

MRI data were obtained using a 3.0-Tesla scanner with a three-dimensional, fast-spoiled, gradient-recalled acquisition at steady state (3D-FSPGR). The following parameters were used: repetition time, ms/echo time, ms/inversion time, 10/4.1/700; flip angle, 10°; field of view, 24 cm; slice thickness, 1.2 mm; resolution, 0.9 × 0.9 × 1.2 mm. All images were corrected for image distortion due to gradient non-linearity using “GradWarp” [18] and for intensity inhomogeneity using “N3” [19]. Image processing for VBM, a fully automatic technique for computational analysis of differences in regional brain volume throughout the brain, was conducted using SPM8 software (Statistical Parametric Mapping 8; Institute of Neurology, London, UK) [20, 21]. The 3D-FSPGR images in native space were spatially normalized; segmented into GM, white matter, and cerebrospinal fluid images; and modulated using the Diffeomorphic Anatomical Registration Through Exponential Lie Algebra (DARTEL) toolbox in SPM8 [22]. The DARTEL was proposed by Ashburner as an alternative method for normalization in the SPM package [20]. To preserve the gray and white matter volumes within each voxel, we modulated the images using the Jacobean determinants derived from the spatial normalization by DARTEL. The resulting modulated GM images were smoothed using an 8-mm full width at half maximum Gaussian kernel according to the recommendation in “VBM Tutorial” written by Ashburner (http://www.fil.ion.ucl.ac.uk/).

### Statistical Analyses

For the analysis of participant demographic and clinical characteristics, unpaired t-tests were performed to compare differences in age, years of education, and proximal IQ between Val/Val individuals and Val/Met individuals. A chi-square unpaired t-test was used to compare differences in sex. Statistical analyses were performed using the statistical software package StatView 5.0 (SAS Institute, Cary, NC). A p-value of <0.05 was assumed to indicate a statistically significant difference.

In the VBM analysis, statistical analyses were performed using the Statistical Parametric Mapping (SPM8) software. Morphological differences in GM between Val/Val individuals and Val/Met individuals were assessed using an unpaired t-test. Age, sex, years of education, and total GM volume were included as covariates of no interest into all analyses to control for potential confounding variables. These analyses yielded statistical parametric maps [SPM (t)] based on a voxel-level height threshold of p < 0.001. We used the topological false discovery rate (FDR) correction in SPM8, which means that the inference on clusters was based on cluster size, controlling the fraction of false positive clusters from among all clusters on average [23, 24]. The significance level was set at FDR-corrected p < 0.05.

## Results

### Demographic and Clinical Data

There were no significant differences between Val/Val individuals and Val/Met individuals with regard to the distributions of sex, age, years of education, and estimated IQ assessed using the Japanese version of National Adult Reading Test [25] (Table 1).

**Table 1:** Demographic Characteristics of Participants.

	Val/Val (n = 27)	Val/Met (n = 22)	p value
Age, mean (range, SD)	41.4 (20–65, 12.0)	40.6 (22–61, 10.3)	0.81
Female, numbers	8	4	0.35
Age of female, mean (range, SD)	48.1 (27–59, 16.7)	35.8 (22–54, 14.1)	0.22
Years of education, mean (SD)	16.8 (3.1)	15.9 (2.3)	0.24
Proxy IQ (SD)	107.8 (9.5)	109.0 (11.1)	0.77

### Morphological Changes in VBM analysis

**Fig 2:**
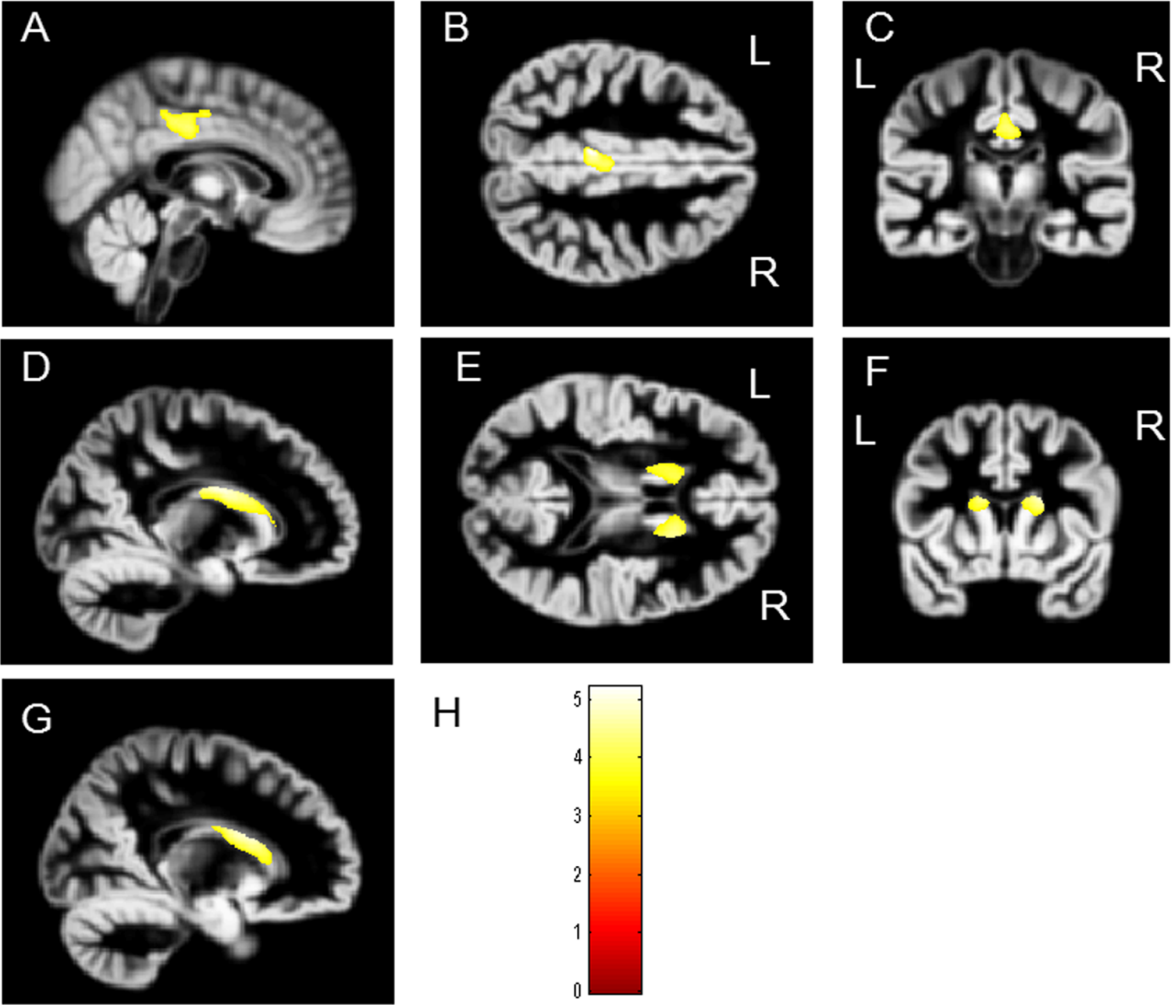
Clusters of significantly decreased gray matter volume in Val/Val individuals compared with that in Val/Met individuals. The SPM {t} is displayed onto the Diffeomorphic Anatomical Registration through Exponentiated Lie Algebra (DARTEL) template of gray matter. The volumes of the bilateral caudate and posterior cingulate cortex (PCC) were significantly smaller in Val/Val individuals than in Val/Met individuals (yellow areas). A-C: The sagittal, axial, and coronal images of the PCC. D: The sagittal image of the right caudate. E-F: The axial and corneal images of the bilateral caudate. G: The sagittal image of the left caudate. H: The color bar indicates the T-value.

The effects of the differences in GM volume between genotypes are shown in Fig 2. The volume of the bilateral caudate and posterior cingulate cortex (PCC) was significantly smaller in Val/Val individuals than in Val/Met individuals (right caudate: FDR-corrected p = 0.048, left caudate: FDR-corrected p = 0.048, and bilateral PCC: FDR-corrected p = 0.048) (Fig 2) (Table 2). In contrast, the volume of the right temporal gyrus and fusiform gyrus were smaller in Val/Met individuals than in Val/Val individuals (temporal gyrus: uncorrected p = 0.002 and fusiform gyrus: uncorrected p = 0.015); however, no voxels survived after FDR correction was applied (temporal gyrus: FDR-corrected p = 0.054 and fusiform gyrus: FDR-corrected p = 0.208).

**Table 2:** Results of Voxel-based morphometry analysis.

Anatomical regions	FDR-corrected p	uncorrected p	Cluster size	T-value	MNI coordinates		
	(culuster level)	(culuster level)		(Voxel level)	x	y	z
**Larger gray matter volume in Val/Met individuals**							
Bilateral posterior cingulate cortex	0.048	0.000	2386	5.21	-6	-32	41
				4.37	-2	-14	46
				4.27	2	-23	37
Right caudate	0.048	0.000	2291	4.79	20	4	21
				4.77	19	13	19
Left caudate	0.048	0.000	2120	5.16	-13	-1	24
				3.39	-16	25	7
**Larger gray matter volume in Val/Val individuals**							
Right superior temporal gyrus	0.054	0.002	2484	5.71	49	-12	2
				4.69	59	-4	-1
				4.65	63	4	2
Right fusiform gyrus	0.208	0.015	1376	5.09	50	-18	-22
				4.49	57	-15	-30

Val = Valine; Met = Methionine; MDD = Major depression disorders; HS = healthy subjects; COMT = Catechol-O-methyltransferase, FDR = false discovery rate, MNI = Montreal Neurological Institute

## Discussion

Previously, the comparison between Val/Val individuals and Met carriers or the linear regression model has contributed to the identification of COMT genotype effects on the brain morphology or function [4–8]. However, based on the non-linear COMT genotype effects [7, 17], the comparison between Val/Val individuals and Met carriers might obscure the difference between Val/Val and Val/Met individuals. The linear regression model based on the linear COMT genotype effects might also be difficult to detect the non-linear COMT genotype effects. Therefore, our analyses by the direct comparison between Val/Val and Val/Met individuals may provide a new finding of the COMT genotype effect on the brain volume.

In this study, the application of whole-brain VBM demonstrated that Val/Val individuals have smaller volumes in the bilateral caudate and PCC than that in Val/Met individuals. Although the COMT genotype is known to affect striatum dopamine levels [26–28], no study has reported differences in striatal morphology between COMT genotypes. Therefore, to the best of our knowledge, this is the first report demonstrating that genetic variation in COMT impacts caudate volume in healthy subjects. In terms of PCC, a previous study also found that the COMT genotype impacts PCC volume in healthy subjects, with Val/Val individuals having a decreased PCC volume compared with that in Met carriers (Val/Met and Met/Met individuals) [6]. Our result of the PCC may be consistent with this previous study, in which the difference between ValVal individuals and Met carriers might be mainly affected by the difference between Val/Val and Val/Met individuals because their subject populations was mainly occupied with Val/Val individuals (n = 141) and Val/Met individual (n = 131), compared to the small number of Met/Met individuals (n = 30).

**Fig 3:**
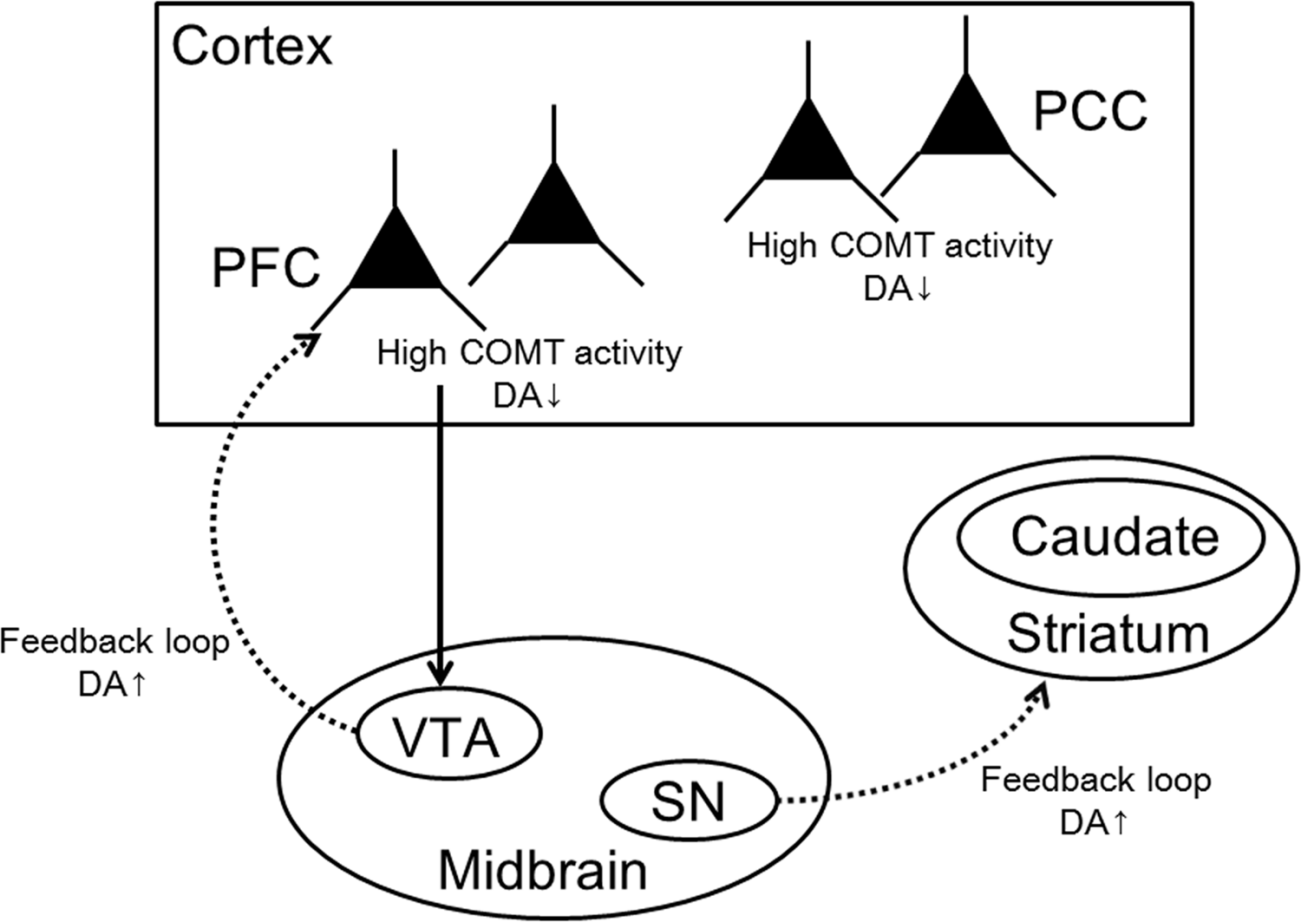
Circuitry elements in the effect of the Val/Val genotype on dopamine levels. Relative to the Val/Met genotype, the COMT enzyme from the Val/Val genotype leads to reduced dopamine levels in the cortex, including the PFC and PCC. Although projections from the PFC to the midbrain directly contact dopaminergic cell groups in the ventral tegmental area that project back to the PFC to generate low dopamine levels, they indirectly elevate the dopamine levels via dopaminergic cell groups in the substantia nigra that project to the striatum [29, 30]. In the striatum, the direct influence of COMT is weak because the dopamine level is predominantly regulated by dopamine reuptake through presynaptic transporters [31, 32]. Therefore, Val/Val individuals have lower dopamine levels in the PCC and higher dopamine levels in the caudate than those in Val/Met individuals. DA = dopamine, PFC = prefrontal cortex, PCC = posterior cingulate cortex, VTA = ventral tegmental area, and SN = substantia nigra.

Our results on brain morphometric differences between COMT genotypes may be related to the effects of varying dopamine levels. A human postmortem study suggested that dopamine levels were higher in the striatum, including the caudate, in Val/Val individuals than in Val/Met individuals [28]. Furthermore, there are previous reports that the COMT genotype has inconsistent effects on dopamine levels between the cortex, including the PCC and striatum [29–32] (Fig 3). Thus, Val/Val individuals have higher dopamine levels in the caudate and lower dopamine levels in the PCC than that in Val/Met individuals.

The current study demonstrated that Val/Val individuals have a smaller volume in both the caudate and PCC than that in Val/Met individuals, although previously reported results on dopamine levels demonstrated an inverse relationship between the PCC and caudate. Our results are supported by previous studies which showed that both over- and under-stimulation with dopamine results in impaired neuronal survival and growth, implying that an optimum range of dopamine levels may exist for maintaining structural integrity [33–37]. Some studies have reported that lower dopamine levels impaired neuronal differentiation [35] because dopaminergic connections coincide with neuronal growth and differentiation [33], and dopamine induces brain-derived neurotropic factor expression, which is the most prevalent growth factor in the central nervous system [34]. In contrast, an excess of extracellular dopamine was also reported to impair neuronal integrity and survival [36]. In an animal study, knockout mice lacking dopamine transporter expression, which is chronically elevated in response to extracellular dopamine levels, showed reduced brain-derived neurotropic factor gene expression [37]. Therefore, we speculate that, in Val/Val individuals, the higher dopamine levels in the caudate and lower dopamine levels in the PCC were both related to the volume reductions in these regions.

This study had several limitations. First, the study was limited by a small sample size, which prevented the exploration of potentially relevant interactions, such as evaluation of other genotypes that may influence brain volume. Further, a small sample size might have led to positive publishing bias. The previous larger sample studies [6, 7], which evaluated the COMT genotype effect including the difference between Val/Val and Val/Met individuals, did not find the effect on the caudate. The discrepancy is likely due to differences regarding the subject populations. In future, larger study is required to confirm our findings. Second, we could not confidently exclude the potential effects of age and gender on the current results, although both age and gender were set as covariates in the VBM analysis. We included subjects with a wide range of age, although some previous studies recruited subjects with a narrow range of age [6, 9]. Further, limited in female, the mean age was not well matched. Previous studies have suggested the possibility that age and gender were related to differences in the effects of COMT genotypes, with COMT activity reported to be the highest at earlier ages and in particular, from 6 to 20 years [17, 38]; and female hormones, such as estrogen, possibly downregulating COMT expression [39]. Third, we did not evaluate the influence of the PCC and caudate morphological change identified in this study on brain function. Future studies evaluating larger numbers of subjects are needed to confirm the influence of the COMT genotype on phenotypic differences.

In conclusion, our study demonstrates that interacting COMT functional variants affect GM regional volumes in healthy subjects. The volume of the bilateral caudate and PCC were smaller in Val/Val individuals than in Val/Met individuals, which may be related to differences in dopamine levels.
